# Development of Hydrogels Fabricated via Stereolithography for Bioengineering Applications

**DOI:** 10.3390/polym17060765

**Published:** 2025-03-14

**Authors:** Youngjin Jeon, Minji Kim, Kwang Hoon Song

**Affiliations:** 1 Department of Nano-Bioengineering, Incheon National University, 119, Academy-ro, Yeonsu-gu, Incheon 22012, Republic of Korea; jyjqqqqq@naver.com (Y.J.); alswlox@naver.com (M.K.); 2 Research Center of Brain-Machine Interface, Incheon National University, 119, Academy-ro, Yeonsu-gu, Incheon 22012, Republic of Korea

**Keywords:** hydrogels, stereolithography, bioprinting

## Abstract

The architectures of hydrogels fabricated with stereolithography (SLA) 3D printing systems have played various roles in bioengineering applications. Typically, the SLA systems successively illuminated light to a layer of photo-crosslinkable hydrogel precursors for the fabrication of hydrogels. These SLA systems can be classified into point-scanning types and digital micromirror device (DMD) types. The point-scanning types form layers of hydrogels by scanning the precursors with a focused light, while DMD types illuminate 2D light patterns to the precursors to form each hydrogel layer at once. Overall, SLA systems were cost-effective and allowed the fabrication of hydrogels with good shape fidelity and uniform mechanical properties. As a result, hydrogel constructs fabricated with the SLA 3D printing systems were used to regenerate tissues and develop lab-on-a-chip devices and native tissue-like models.

## 1. Introduction

**Figure 1:**
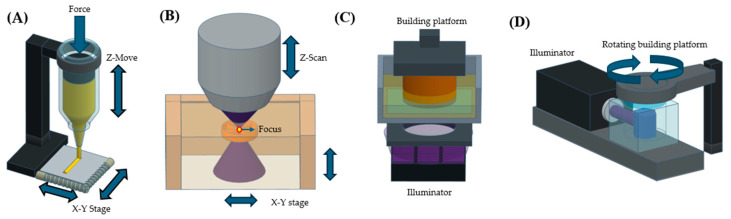
Schematic illustrations of various 3D printing systems that can utilize hydrogel precursors as 3D printing inks. Working principles of extrusion-(**A**), two-photon polymerization-(**B**), stereolithography (SLA)-(**C**) and volumetric-type (**D**) 3D printing systems.

Hydrogels that could recapitulate characteristics of the extracellular matrix (ECM) have been used for bioengineering applications [1,2,3,4,5,6]. Indeed, hydrogels exhibiting ECM-like features (degradability [7,8], stiffness [9,10], adhesiveness to cells [11,12] and structures [13]) have been useful for those applications. In particular, hydrogels with native environment-like structures were developed as scaffolds to regenerate tissues [14,15,16], lab-on-a-chip devices [17,18,19] and native tissue-like models [20,21,22,23]. In previous studies, photolithography [24], molding [25,26], electrospinning [27] and 3D printing [28] techniques were exploited for the fabrication of hydrogels with distinct structures. Particularly, 3D printing techniques involving the extrusion [17,29], two-photon polymerization [30], stereolithography (SLA) [31] and volumetric [32] types typically allowed fabrication of complex structures, although the hydrogel precursors, the main materials used to fabricate hydrogels, were required to exhibit properties appropriate for the 3D printing types (Figure 1). Specifically, hydrogel precursors with relatively high viscosity were required for extrusion-type 3D printing systems because the high viscosity of the precursors helped to maintain printed structures (Table 1) [33,34]. On the other hand, hydrogel precursors with low viscosity could be useful for the fast fabrication of hydrogels in SLA systems by rapidly filling the gaps between the fabricated structures and reservoir bottom with the precursors. Hydrogel precursors that could be polymerized with light exposure have been also used for two-photon polymerization-, stereolithography- and volumetric-type 3D printing systems. Light exposure to a defined space of precursors in the two-photon polymerization, SLA and volumetric type 3D printing allowed the fabrication of hydrogels in various structures with good shape fidelity. There could be limitations in the fabricable structures of hydrogels, varied by 3D printing types. In extrusion-type 3D printing, the hydrogel precursors were deposited within a shear-thinning support bath for the fabrication of overhanging structures [33]. Overhanging structures that are too long might not be fabricated using SLA 3D printing systems [35]. The depth of light penetration through the precursors in two-photon polymerization 3D printing also could limit the thickness of overall hydrogel structures [36,37]. There are also advantages for using the SLA systems for the fabrication of hydrogels. Compared to the two-photon polymerization type, the SLA types were cost-effective, while maintaining relatively good shape fidelity [38]. Additionally, the SLA type could offer better layer thickness control and structure precision with more uniform mechanical properties than the volumetric types that could cure an entire volume of the precursors simultaneously [39].

**Figure 2:**
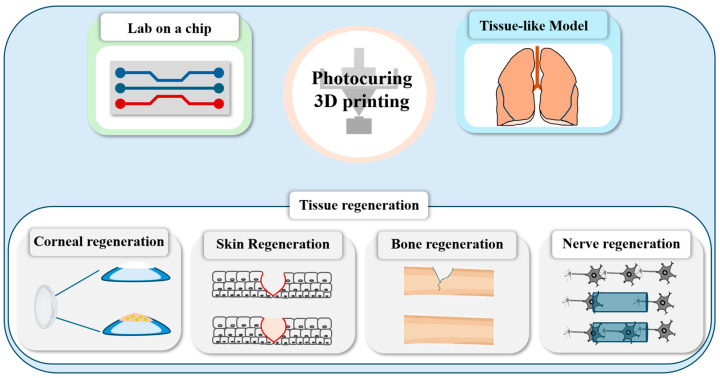
Schematic illustrations describing the bioengineering applications of hydrogel constructs prepared via SLA-based 3D printing systems. Specifically, the hydrogel constructs were used for regeneration of cornea, skin, bone and nerve and development of lab-on-a-chip devices and tissue-like models.

In this review, we have focused on the composition of hydrogel precursors used for SLA-type 3D printing, the working mechanisms and types of the SLA systems and the bioengineering applications of hydrogel constructs fabricated with SLA 3D printing. As bioengineering applications of them, we have summarized the research that utilized hydrogels fabricated via the SLA 3D printing for the regeneration of tissues (nerve, bone, skin and cornea) and development of lab-on-a-chip devices and native tissue-like models (Figure 2). We believe that information on SLA 3D printing techniques and the hydrogel precursors for them will be useful for bioengineering fields and can help to expand their applications to practical uses.

## 2. Materials and Methods

### 2.1. Materials

Hydrogels with user-defined structures have been fabricated with diverse 3D printing techniques, such as extrusion-based 3D printing [29], stereolithography (SLA) [31], two-photon polymerization [30] and volumetric 3D printing [32]. In particular, SLA allowed the relatively rapid generation of hydrogels in complex structures with good printing fidelity. Typically, the SLA technique generates overall hydrogel structures by crosslinking layers of hydrogel precursors via light illumination and successively accumulating the crosslinked layers. Due to this working principle, hydrogel precursors that can be crosslinked through light exposure have generally been used for the SLA processes. For instance, GelMA [15], PEGDA [45] and HEMA [46] have been used as major components of hydrogel precursors during the SLA processes (Table 2). In addition, LAP [47,48], Irgacure 2959 [49] and Irgacure 651 [50], as photo-initiators that could trigger crosslinking the hydrogel precursors via light exposure, were involved in the hydrogel precursors (Table 3). The hydrogel precursors also included photo-absorbers such as Tartarzine [20,51] and TEMPO [52] to control the thickness of the crosslinked layers (Table 4). The thickness of crosslinked layers can be determined by light penetration controlled by concentration of the photo-absorbers in the hydrogel precursors.

Hydrogel precursors involving these components for the SLA processes were required to exhibit low viscosity and light-mediated polymerization. Indeed, the precursors need to rapidly fill the gaps formed by lifting printed hydrogels from the bottom surfaces of reservoirs. High viscosity might delay filling the gaps with the precursors, resulting in defects of printing fidelity [53]. In addition, hydrogels were formed via light-mediated polymerization of the precursors in the SLA processes. Thus, the main polymers involved in the hydrogel precursors typically contained functional groups such as methacrylate and norbornene for light-mediated polymerization [40].

**Table 2:** Types of main materials within hydrogel precursors used as inks for SLA-based 3D printing systems.

Material	Chemical Structure	Characteristics
Gelatin Methacrylate (GelMA)	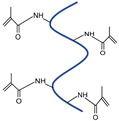	Adhesive to cells [54,55], biodegradable [54,55]
Polyethylene Glycol Diacrylate (PEGDA)	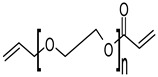	Stable after printing [56,57], excellent printing fidelity [56,57]
Poly(Glycerol Sebacatemethacrylate) (PGS)	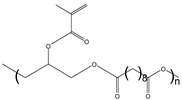	Native tissue-like Young’s modulus [58], highly flexible [58], stable under PBS [58], degradable by lipase [58]
Acrylamide (AAm)	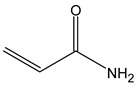	High water absorption [59], stability under stress [59,60]
2-Hydroxyethyl Methacrylate (HEMA)	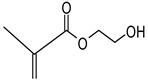	Low immunogenicity [61], similar Young’s modulus to cartilage [46,61,62]
Acrylic Acid (AA)	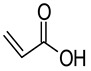	Anionic [59], pH-sensitive [59], ion-exchange capability [59], high water absorption [59]
Hyaluronic Acid-Norbornene (HA-NB)	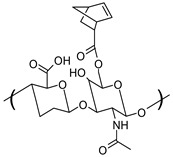	Bioadhesive [63], promotes cell adhesion and proliferation [63], Thiol-ene reaction [63,64]
Pluronic F127 Diacrylate (F127DA)	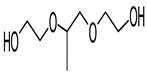	Maintaining long-term structural stability in a PBS environment [58], high stretchability [64], cytocompatibility [64,65]

**Table 3:** Types of photo-initiators involved within hydrogel precursors used as inks for SLA-based 3D printing systems.

Photo-Initiator	Wavelength of Absorbable Light	Chemical Structure	Ref.
Lithium phenyl (2,4,6-trimethylbenzoyl) phosphinate (LAP)	375 nm	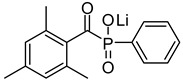	[66,67]
2-Hydroxy−4′-(2-hydroxyethoxy)−2-methylpropiophenone (Irgacure 2959)	257 nm	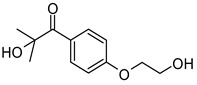	[68,69]
2′,4′,5′,7′-Tetrabromofluorescein disodium salt (Eosin y)	514 nm	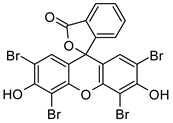	[70]
Tris(2,2′-bipyridyl)dichlororuthenium(II) hexahydrate/sodium persulfate(Ru/SPS)	405 nm	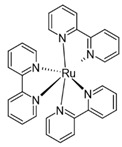	[71,72]
Water-soluble 2,4,6-trimethylbenzoyl-diphenylphosphine oxide (TPO) nanoparticle	365 nm	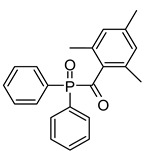	[73,74,75]
Phenyl bis(2,4,6-trimethylbenzoyl)phosphine oxide (Irgacure 819)	405 nm	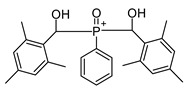	[76,77]
2-Hydroxy-2-methylpropiophenone (HMPP)	365 nm	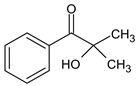	[58,78]

**Table 4:** Types of photo-absorber involved within hydrogel precursors used as inks for SLA-based 3D printing systems.

Photo-Absorber	Wavelength of Absorbable Light	Chemical Structure	Ref.
Tartrazine (acid yellow 23)	425	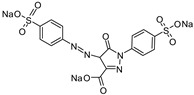	[20,69,79,80,81]
Sudan 1	481	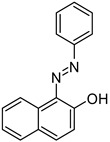	[74,82]
Quinoline yellow	415	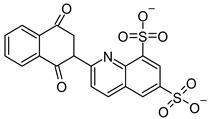	[63,83]
Orange G	480	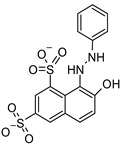	[84,85]
Phenol red	422,559	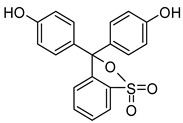	[63]

### 2.2. Methods

**Figure 3:**
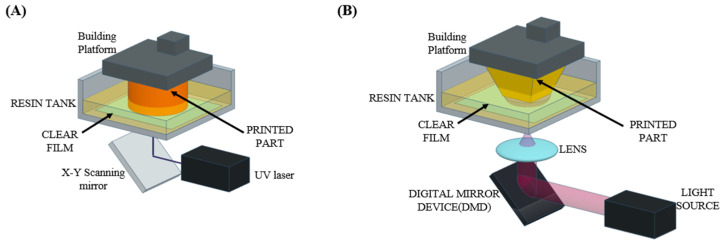
Schematic illustrations describing the working principles of SLA-based 3D printing systems. Working principles of laser-based SLA systems (**A**) and digital light processing (DLP)-based SLA systems (**B**).

The SLA techniques generally utilize either point-scanning systems to fabricate a layer of hydrogels via scanning the hydrogel precursor with a focused light exposure (laser-based SLA) or a digital micromirror device (DMD) system to cure an entire layer of the hydrogel precursor at once with the exposure of a light pattern onto the precursor (DLP-based SLA) (Figure 3). SLA techniques exploiting the point-scanning system typically allow the fabrication of hydrogels with better printing fidelity but require more time to fabricate a layer of hydrogel compared to the DLP-based SLA techniques (Table 5). Using either of them, the first hydrogel layer can be fabricated on a specimen holder and then the rest of the hydrogel layers can be fabricated on the previously fabricated ones to eventually obtain hydrogels with the desired 3D designs.

In these SLA processes, factors such as the intensity, wavelength and exposure time of light and the layer thickness need to be considered. The intensity and wavelength of light could affect the degree of polymerization, resulting in varied mechanical properties for the fabricated hydrogel constructs [53,59]. Insufficient exposure times might cause incomplete precursor polymerization [86], while excessive light exposure times might reduce the porosity of hydrogels [86]. Additionally, the resolution of SLA processes and stability of hydrogel structures could be determined by layer thickness [87]. It would be challenging to fabricate small features by successively generating thick layers. In contrast, the fabrication of hydrogels with thin layers might require a large amount of time for the overall processes, although it provides good shape fidelity for the fabrication of hydrogel constructs.

**Table 5:** Characteristics and applications of laser-based and DLP-based SLA systems.

SLA Type	Characteristics	Applications
Laser-based SLA	Very high 3D printing resolution, slow 3D printing processes, point polymerization.	soft robotics [88], neural stem cell proliferation [89]
DLP-based SLA	Faster 3D printing processes, 3D printing resolution can be lower than laser-based SLA, whole layer polymerization	sciatic nerve regeneration [90] osteosarcoma cell proliferation [91], cartilage tissue engineering [92]

## 3. Applications

### 3.1. Nerve Regeneration with Hydrogels Fabricated via SLA

**Figure 4:**
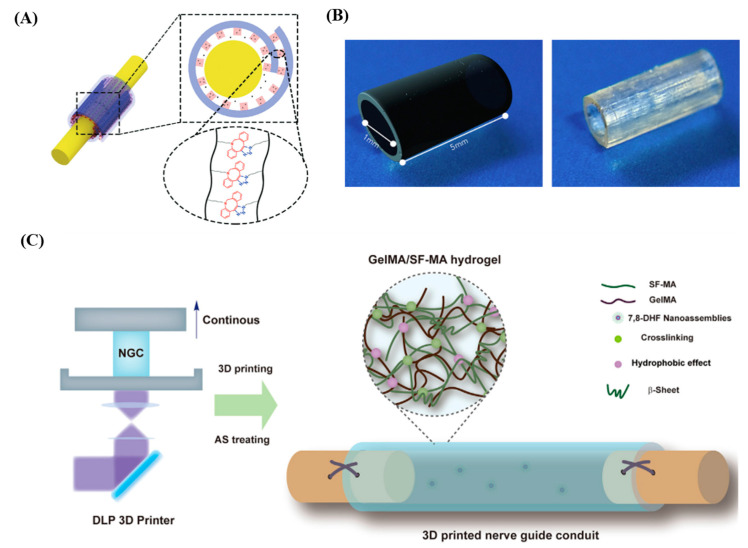
Nerve tissue regenerations using hydrogel constructs prepared via SLA-based 3D printing systems. (**A**) A schematic illustration of self-adhesive bandages used for repair of transected sciatic nerves. The bandages are self-adhesive via the click reaction of -N3 and -DBCO [90]. Distributed under the Creative Commons Attribution License 4.0 (CC BY, https://creativecommons.org/licenses/by/4.0/ (accessed on 1 February 2025)). The bilayered bandages were rolled and adhered to themselves via the click reaction to finally wrap the nerves with tube structures. (**B**) A design and a picture of a conduit to guide nerves [58]. Distributed under the Creative Commons Attribution License 4.0 (CC BY, https://creativecommons.org/licenses/by/4.0/ (accessed on 1 February 2025)). The nerve guidance conduits were used for the regeneration of trimmed common fibular nerves. (**C**) Schematic illustrations of a DLP-based 3D printing system used for the fabrication of nerve guide conduits and the nerve guide conduit configurations [93]. Distributed under the Creative Commons Attribution License 4.0 (CC BY-NC-ND 4.0, https://creativecommons.org/licenses/by-nc-nd/4.0/ (accessed on 1 February 2025)). The conduits were used to regenerate sciatic nerves.

Previously, hydrogel implants fabricated using GelMA [90], Poly(Glycerol Sebacate Methacrylate)(PGSm [58] and GelMA/SF-MA [93] techniques have been used to regenerate various nerve tissues and were indeed effective in the regeneration of nerves. In particular, hydrogel constructs fabricated with SLA techniques were developed as bandages for wrapping injured nerves [90] and nerve conduit grafts guiding nerve growth [94] for the regeneration of damaged nerves. For instance, self-adhesive bandages in a bilayer structure were fabricated with a DLP-based SLA technique and used to regenerate transected sciatic nerves in Sprague Dawley (SD) rat models (Figure 4A). Specifically, the bandages were prepared by forming grating layers with azide-functionalized gelatin methacrylate (N3-GelMA) and methoxypolyethylene glycol-poly(ε-caprolactone) (MPEG-PCL) nanoparticles containing XMU-MP-1 on basal layers fabricated with dibenzyl cyclooctyne-functionalized gelatin methacrylate (DBCO-GelMA) in a rectangular shape. For the regeneration of the transected nerves, the nerves treated with neurorrhaphy were wrapped with the bandages, and XMU-MP-1 (a nerve-regenerating drug) loaded in the grating structures (inner layer) was released. The wrapped bandages were secured via click-reaction-mediated adhesion at overlapped regions. Indeed, the transected sciatic nerves treated with neurorrhaphy and wrapped with the bandages carrying the nerve-regenerating drug were effectively restored.

Nerve guidance conduits (NGCs; length: 5 mm, inner diameter: 700 µm and wall thickness: 350 µm) that were flexible and degradable were also fabricated with poly(glycerol sebacate methacrylate) (PGSm) via the DLP-based SLA technique and used to treat trimmed common fibular nerves in mice (Figure 4B). As a treatment, the trimmed nerve endings were inserted into the aforementioned NGCs and secured with a fibrin glue. As a result, axons were regenerated through the NGSs, and the nerve regeneration using NGCs was equivalent to graft-mediated repair of the trimmed nerves. Damaged sciatic nerves in rat sciatic nerve injury models were also regenerated by implanting hydrogel conduits delivering 7,8-dihydroxyflavone (7,8-DHF), a TrkB receptor agonist that could support axonal growth and remyelination (Figure 4C). A conduit with outer and inner diameters of 1.7 mm and 1.1 mm, respectively, was fabricated with 7,8-DHF, gelatin methacryloyl (GelMA) and silk fibroin methacrylate (SF-MA) via DLP-based SLA techniques and implanted to connect the dissected sciatic nerves of the injury models by inserting the nerves into the conduits and suturing them. The implantation of the hydrogel conduits promoted the recovery of sciatic nerves and their functions and suggested a potential approach to treat long-gap injuries in nerves.

The mechanical properties, degradation rates and cell viability of the hydrogels were analyzed to demonstrate that hydrogel constructs were useful for nerve regeneration (Table 6). For instance, measurements of loss modulus and tensile strength demonstrated self-adhesive capability and durability, respectively, for nerve repair applications (Figure 4A). Also, Schwann cell viability after 72 h was 95.4%, suggesting excellent biocompatibility. A Young’s modulus of 3.2 MPa demonstrated that PGSm could mimic the mechanical properties of native nerve tissue, and an elongation at break of 220 ± 14% showed that it could be used for nerve conduit applications (Figure 4B). The GelMA-to-SF-MA ratio determined the tensile modulus of the hydrogel, ranging from 0.027 MPa for GelMA only to 3.51 MPa for SF-MA only (Figure 4C). The viability of Schwann cells on a GelMA/SF-MA 1:1 ratio and GelMA only was 91.2 and 83.5%, respectively, indicating that the inclusion of SF-MA increased cell viability.

### 3.2. Bone Regeneration with Hydrogels Fabricated via SLA

**Figure 5:**
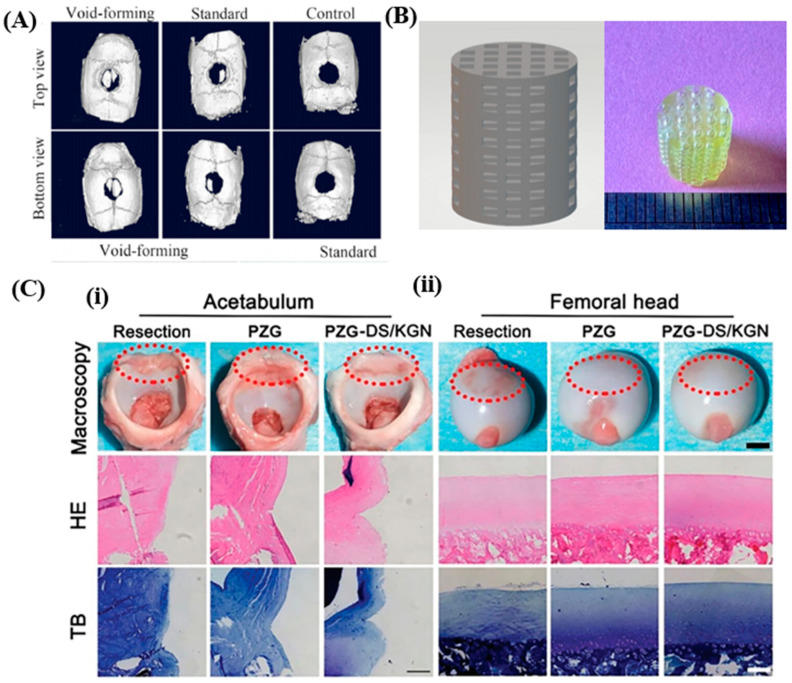
Bone tissue regenerations using hydrogel constructs prepared via SLA-based 3D printing systems. (**A**) Micro-CT images showing parietal bone regeneration of void-forming hydrogel (hydrogel with pores)-treated, standard hydrogel (hydrogel without pores)-treated, and control (non-treated) groups [60]. Distributed under the Creative Commons Attribution License 4.0 (CC BY-NC-ND 4.0, https://creativecommons.org/licenses/by-nc-nd/4.0/ (accessed on 1 February 2025)). (**B**) A design and a picture of a scaffold used for the regeneration of femoral bone [65]. Distributed under the Creative Commons Attribution License 4.0 (CC BY, https://creativecommons.org/licenses/by/4.0/ (accessed on 1 February 2025)). The scaffolds were fabricated via a DLP-based SLA technique. (**C**) Pictures and hematoxylin and eosin (HE) and toluidineblue (TB) images of the resection (implanted without scaffolds), PZG (implanted with hydrogel scaffolds only) and PZG-DS/KGN (implanted with hydrogel scaffolds containing diclofenac (DS) and kartogenin (KGN)) groups [77]. Permit ordered. The experiments were implemented on the acetabulum (**i**) and femoral head (**ii**).

In addition to nerves, bones such as parietal bone [60], femoral bone [65] and cranial dorsal labrum [77] were regenerated with hydrogel constructs fabricated via SLA techniques. For instance, porous GelMA hydrogels containing bone mesenchymal stem cells (BMSCs) were used to repair full-thickness craniotomy at the parietal bone of rats (Figure 5A). The porous hydrogels were formed using a hydrogel precursor containing GelMA, dextran microdroplets and BMSCs with a DLP system and removing the dextran from the GelMA hydrogels containing BMSCs. Compared to the control (the parietal bone defect models without any treatment) and standard (the parietal bone defect models treated with GelMA hydrogels with BMSCs and without pores) groups, the void-forming group (the parietal bone defect models treated with porous GelMA hydrogels containing BMSCs) showed the most parietal bone regeneration. For the regeneration of femoral bone defects, the DLP technique was also used to fabricate scaffolds in lattice structures with GelMA, polyethylene glycol diacrylate (PEGDA), and pluronic F127 diacrylate (F127DA) as basic materials (Figure 5B). The scaffolds promoted the osteogenic differentiation of mesenchymal stem cells (rBMSCs) in an osteoinductive environment and eventually enhanced regeneration of femoral bones when they were implanted into the femoral bone defects of rabbit models. Hydrogel constructs fabricated using the DLP technique were used as scaffolds to repair defects at the cranial dorsal labrum and lateral meniscus of porcine models (Figure 5C). For fabrication of the hydrogel scaffolds, emulsified mixtures containing acrylic amide (AAm, hydrophilic monomer), 2-acrylamido-2-methyl-1-propanesulfonic acid (AMPS, hydrophilic monomer), isobornyl acrylate (IBOA, hydrophobic monomer), bis(2,4,6-trimethylbenzoyl) phenyl phosphine oxide (PI819, hydrophobic photo-initiator) and waterborne polyurethane acrylate (WPUA) were used as a DLP printing ink. As a result, both hydrophilic and hydrophobic drugs could be loaded within the fabricated hydrogel scaffolds. Compared to groups implanted with nothing (denoted as resection) and the hydrogel scaffold only (denoted as PZG), the group implanted with the hydrogel scaffolds containing diclofenac (DS; hydrophilic drug) and kartogenin (KGN; hydrophobic drug) (denoted as PZG-DS/KGN) exhibited the most regeneration of the cranial dorsal labrum and lateral meniscus.

The mechanical properties, degradation rates and cell viability of the hydrogels used in the aforementioned studies showed the various features of the hydrogels (Table 7). The viability of encapsulated bone marrow stem cells, which was over 90% after 7 days of culture, demonstrated the biocompatibility of hydrogels (Figure 5A). Additionally, the degradation study revealed that GelMA/Dextran hydrogels degraded with collagenase. The compressive moduli of PF (10 *w/v*% PEGDA/5 *w/v*% F127DA) and GPF (5 *w/v*% GelMA/10 *w/v*% PEGDA/5 *w/v*% F127DA) were 127.4 ± 12 kPa and 92.34 ± 6.80 kPa, respectively (Figure 5B). The tensile strength of hydrogel was 9.9 MPa, and the compressive modulus of the hydrogels was similar to native labrum property (Figure 5C).

### 3.3. Skin and Cornea Regeneration with Hydrogels Fabricated via SLA

**Figure 6:**
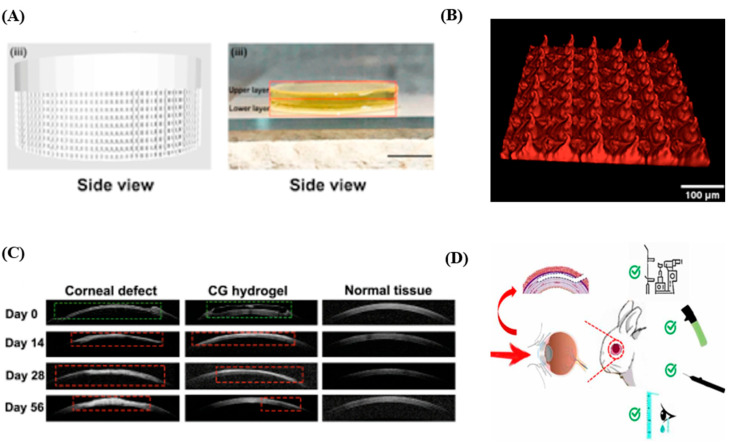
Skin and corneal tissue regenerations using hydrogel constructs prepared via SLA-based 3D printing systems. (**A**) A side view computer-aided design (CAD) image and a side view picture of a scaffold used to treat skin defects [63]. Permit ordered. The skin scaffolds were prepared with a DLP-based 3D printing system. Scale bar: 2 mm. (**B**) An image of a hydrogel matrix mimicking the dermoepidermal junction, obtained with two-photon microscopy [95]. Distributed under the Creative Commons Attribution License 4.0 (CC BY-NC 3.0, https://creativecommons.org/licenses/by-nc/3.0/ (accessed on 1 February 2025)). Scale bar: 100 µm. (**C**) Corneal defect, CG hydrogel and normal tissue group images obtained with anterior segment optical coherence tomography (AS-OCT) [69], Distributed under the Creative Commons Attribution License 4.0 (CC BY, https://creativecommons.org/licenses/by/4.0/ (accessed on 1 February 2025)). The green and red frames indicate defect areas and scar areas, respectively. (**D**) Schematic illustrations describing postoperative examinations on intrastromal keratoplasty (ISK) models [96]. Distributed under the Creative Commons Attribution License 4.0 (CC BY, https://creativecommons.org/licenses/by/4.0/ (accessed on 1 February 2025)).

A bioink containing gelatin methacrylate (GelMA), N-(2-aminoethyl)-4-(4-(hydroxymethyl)-2-methoxy-5-nitrosophenoxy) butanamide (NB)-linked hyaluronic acid (HA-NB) was used to develop functional living skins (FLSs) via the DLP technique; the FLSs were used to repair skin defects of rat and pig models (Figure 6A). The FLSs were composed of upper layers involving human skin fibroblasts (HSFs), lower layers involving human umbilical vein endothelial cells (HUVECs) and microchannels that facilitate neo-tissue formation. Implantation of the FLSs to full thickness defects of skin at dorsal surfaces promoted the regeneration of skin and skin appendages. Precursors containing GelMA, collagen and either ruthenium or LAP as a photo-initiator were used to fabricate hydrogel constructs mimicking the structures of the dermoepidermal junction, and the stability of the constructs was tested (Figure 6B). The hydrogel constructs mimicking the dermoepidermal junction were fabricated with precursors containing GelMA, collagen and ruthenium via two-photon polymerization (TPP), and large 2D matrixes were fabricated with precursors containing GelMA and collagen LAP via the DLP-based SLA technique. Typically, TPP allows better printing fidelity but requires more time for the printing process than the DLP-based SLA technique. Thus, the time required for 3D printing the large 2D matrixes was saved by using the DLP-based SLA technique, while the dermoepidermal junction structures were prepared with good printing fidelity in this research. The hydrogel constructs implanted to the defects of skin were stable on day 14 without signs of degradation that might allow macrophage infiltration through the constructs. Artificial corneas with smooth double curvatures were implanted to repair cornea defects in rabbit models (Figure 6C). Specifically, the artificial corneas were fabricated with precursors containing corneal decellularized extracellular matrix (CECM) obtained by decellularizing the stroma layers of pig corneas and gelatin methacryloy (GelMA) via the DLP-based SLA technology. After the implantation of the artificial corneas to the cornea defects, the epitheliums of the corneas were almost completely regenerated within 2 weeks. The corneal transparencies were also recovered and were similar to the transparency levels of a normal group (left eyes without defects) when they were compared 2 months after the implantation. Additionally, refractive lenticules designed to treat refractive errors were developed with poly-N-Acryloyl glycinamide (NAGA)-GelMA (PNG) bio-inks via DLP technology (Figure 6D). Indeed, the implantation of the refractive lenticules to intrastromal keratoplasty models of New Zealand white rabbits showed stromal regeneration with controlled ECM production instead of excessive scarring.

The mechanical properties, degradation rates and cell viability of the hydrogels were measured to use them for the regeneration of skin and corneal tissues (Table 8). The Young’s modulus of the FLSs, 30.53 kPa, was similar to the mechanical properties of soft tissues (Figure 6A). The microchannels (200 μm in diameter) involved in the hydrogel constructs also supported the growth of HSFs HUVECs. In addition, stiff hydrogel constructs (Young’s modulus = 191 ± 35 kPa) promoted the proliferation of fibroblasts, while softer hydrogels (Young’s modulus = 37 ± 12 kPa) were less effective for fibroblast proliferation (Figure 6B). The Young’s modulus of the hydrogel was 26.68 kPa, approximately 33.13% higher than GelMA-only hydrogels, which improved mechanical integrity (Figure 6C). The hydrogels mimicked human corneal tissue by exhibiting a Young’s modulus (0.145 MPa) similar to that of human corneal tissue (0.281 ± 0.214 MPa) (Figure 6D). Additionally, the compressive strength (6.9879 MPa) and compressive strain (84.718%) of the hydrogels demonstrated the elasticity and toughness of the hydrogels.

### 3.4. Lab-on-a-Chip Devices Developed with Hydrogels Fabricated via SLA

**Figure 7:**
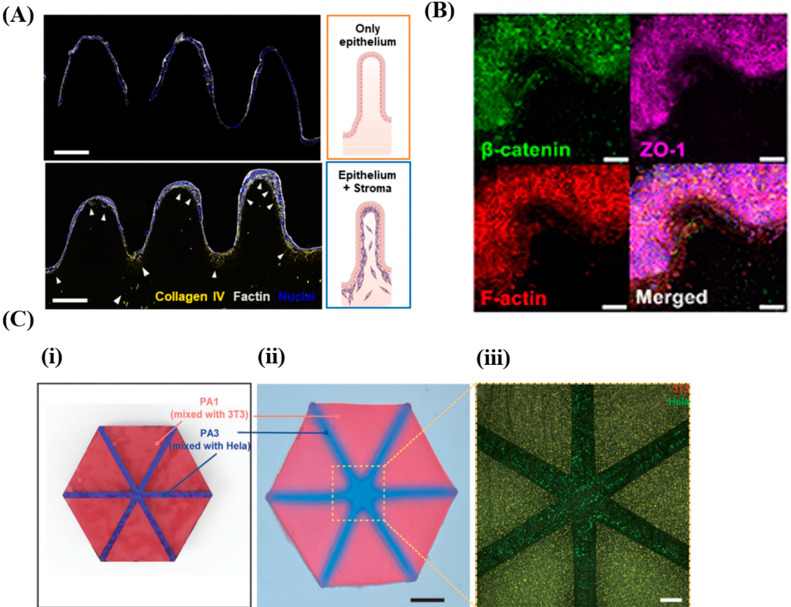
Development of lab-on-a-chip devices exploiting hydrogel constructs prepared via SLA-based 3D printing systems. (**A**) Collagen IV (yellow), F-actin (grey) and nuclei (blue) images to display the distribution of epithelial cells and fibroblasts at crypt–villus structures [81]. Distributed under the Creative Commons Attribution License 4.0 (CC BY, https://creativecommons.org/licenses/by/4.0/ (accessed on 1 February 2025)). Scale bars: 200 μm. (**B**) Maximum intensity projection images of DAPI (blue), β-catenin (green), ZO-1 (magenta) and F-actin (red), obtained by imaging 3T3 fibroblasts and Caco-2 epithelial cells at a part of the hydrogel in villi shape [80]. Distributed under the Creative Commons Attribution License 4.0 (CC BY, https://creativecommons.org/licenses/by/4.0/ (accessed on 1 February 2025)). Scale bar: 100 µm. (**C**) A schematic design (**i**) and an optic image (**ii**) of hydrogel constructs with a hepatic lobular structure [100]. Permitted. The red and blue regions indicate PA1 hydrogels containing 3T3 cells and PA3 hydrogels containing Hela cells, respectively. Scale bar: 2 mm. (**iii**) A fluorescent image of NIH-3T3 cells (red) and Hela-GFP cells (green) within hydrogels in a hepatic lobular shape. Scale bar: 500 μm.

Previously, microfabrication techniques such as molding [97], extrusion-based 3D printing [98] and SLA [99] were applied to the fabrication of hydrogels with structures mimicking native environments and those hydrogels were utilized as the main components of lab-on-a-chip devices. In particular, the SLA technique allowed the fabrication of hydrogels in complex structures with relatively good printing fidelity. For instance, DLP technology was used to fabricate arrays of pillars mimicking crypt–villus structures in intestines and containing fibroblasts, and they were used to develop in vitro intestinal tissue models (Figure 7A). The fibroblasts were encapsulated within the structures by using inks composed of GelMA, PEGDA, tartrazine, LAP and NIH-3T3 fibroblasts for the fabrication, and Caco-2 cells, as epithelial cells, were seeded on the surfaces of the structures. The epithelial cells formed monolayers, with formation of tight junctions on the crypt–villus structure containing fibroblasts working as supportive stroma. Indeed, the junctions of epithelial cells were more tightly formed on the structures containing the fibroblasts than the junctions formed on the structures without the fibroblasts. Similarly, gut-on-chips containing fibroblasts and intestinal epithelial cells with villi structures were fabricated using the DLP system (Figure 7B). The precursors used for fabrication of the chips contained GelMA, PEGDA, LAP and tartrazine as the main materials, and NIH 3T3 cells were also included in the precursors for the fabrication of fibroblast-laden villi structures. Specifically, for gut-on-chips, hydrogel channels involving villi structures and encapsulating 3T3 fibroblast cells were fabricated via the DLP system, and the surfaces of the villi structures were seeded with Caco-2 cells. In other research, inks composed of PEGDA, acrylamide (AAm), LAP and red food dye (as a photo-absorber) were used for the fabrication of hydrogels with complex architectures via the DLP-based SLA system (Figure 7C). The mechanical properties of the hydrogel constructs were altered by varying the PEGDA-AAm concentration of the inks. As a result, cell-laden hydrogel constructs with complex structures and heterogeneity in mechanical properties, such as a simplified hepatic lobule, a hollow tube containing triply periodic minimal surfaces (TPMS) and a vascular network, were fabricated.

The mechanical properties, degradation rates and cell viability of the hydrogels were analyzed to use them for the development of lab-on-a-chip devices (Table 9). The elastic modulus (5.94 ± 0.19 kPa) and storage modulus (2.07 ± 0.41 kPa) of the hydrogels were close to the mechanical properties of intestinal tissue (Figure 7A). The shear stress in hydrogel channels containing villi-like structures was 0.014–0.03 dyn/cm^2^ when the flow rate through the channels was 5–10 µL/min (Figure 7B). The elastic modulus of soft hydrogels (45 kPa) and stiff hydrogels (210 kPa) were used to model various tissue interfaces (Figure 7C).

### 3.5. Native Tissue-like Models Developed with Hydrogels Fabricated via SLA

**Figure 8:**
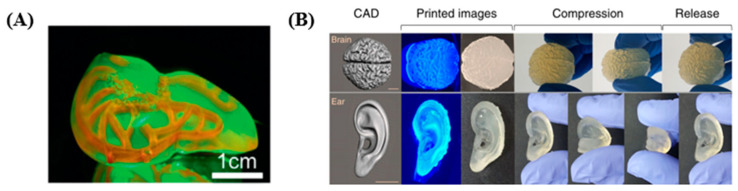
Development of native tissue-like models with hydrogel constructs prepared via SLA-based 3D printing systems. (**A**) A liver model mimicking the exteriors of a liver and involving complex blood vessel-like channels [41]. (**B**) CAD images and pictures of a brain model and an ear model [104]. Distributed under the Creative Commons Attribution License 4.0 (CC BY, https://creativecommons.org/licenses/by/4.0/ (accessed on 1 February 2025)). Those models were intact under compression and recovered their original structures by removing the compression.

The development of 3D printing techniques allowed the fabrication of hydrogels with native tissue-like complex structures. Specifically, hydrogels in hearts [20,101] and vascular network [102] shapes were obtained by depositing hydrogel precursors within a hydrogel support bath via extrusion-type 3D printing systems. Additionally, SLA techniques were used to fabricate bicuspid valve [20], alveolar [20], liver [41], ear [103,104], nose [104], brain [104], trachea [104], heart and lung hydrogel structures. For instance, A DLP-based SLA technique permitting the fast fabrication of hydrogel constructs mainly via the fast flow of hydrogel precursors was used to fabricate PEGDA liver models involving internal channel networks [41] (Figure 8A). In this study, the viability and albumin production of HepG2 cells encapsulated in hydrogel liver models with the perfusion of media through internal channel networks were compared to those of HepG2 cells in hydrogel liver models without internal channel networks. In addition, silk fibroin modified with glycidyl methacrylate (Sil-MA) was used to fabricate the brain, ear, trachea, heart, lung and vasculature hydrogels via a DLP-based SLA system [104] (Figure 8B). The physical and rheological characteristics of the hydrogels could be controlled by variation of the Sil-MA concentration, and Sil-MA was useful for the DLP-based 3D printing system. PEGDA hydrogels with bicuspid valve and alveolar structures were fabricated with a DLP-based SLA system [20]. In this research, the dynamics of the valve leaflets and flow patterns were observed when flows were generated through the bicuspid structures. Additionally, the oxygenation of deoxygenated red blood cells (RBCs) was demonstrated by flowing the deoxygenated RBCs through blood vessel-like channel networks encompassing an air sac ventilated cyclically with oxygen gas.

Likewise, the mechanical properties, degradation rates and cell viability were analyzed for the hydrogel constructs developed for native tissue-like models (Table 10). The elastic modulus of the hydrogels was less than 8 kPa (Figure 8A). The compressive and tensile strength of 30 % Sil-MA hydrogels were 910 kPa and 75 kPa, respectively (Figure 8B).

## 4. Discussion and Future Application

Biofabrication techniques such as photolithography, molding, electrospinning and 3D printing have been developed to meet the need of hydrogels with structures that could play important roles in bioengineering. Typically, photolithography was used to obtain hydrogel constructs via illuminating light patterns to photo-crosslinkable hydrogel precursors [24]. The light patterns used in photolithographic processes could be generated by illuminating light through photo-masks. They could limit shape changes of hydrogel constructs in the height direction, although some photolithography strategies were developed to overcome this issue and fabricate complex structures. Molding is a process to fabricate hydrogel constructs by filling the void space of molds with hydrogel precursors and polymerizing the precursors [25]. It is important to have molds containing well-defined structures, and the overall process typically does not require a large amount of time. Electrospinning techniques use electrical forces to fabricate fibrous hydrogels that can recapitulate the structures of ECMs [27]. Electrospun hydrogel fibers have been used for biomedical applications. Compared to these techniques, manufacturing hydrogels with more complex exterior designs and internal architectures (such as microchannels and pores) are available through 3D printing techniques, although designs could be limited by the types of 3D printing system. In particular, SLA 3D printing techniques have been shown to generate hydrogels with architectures useful for tissue regeneration, the development of lab-on-a-chip devices and relatively large native tissue-like models. The structures of the hydrogels used for these applications can be classified into multi-layer, tube, lattice and dome structures (Table 11). For instance, hydrogels with multi-player, tube, lattice and/or dome structures were fabricated via SLA techniques and were effective in the regeneration of nerve, skin, bone and corneal tissues. Likewise, SLA techniques were used to fabricate hydrogels with multi-layer, lattice and/or channel structures, and they were used to develop lab-on-a-chip devices recapitulating crypt–villus and hepatic lobule structures. Additionally, the complex external or internal architectures of hydrogels were fabricated with SLA techniques and developed as native tissue-like models. SLA techniques have been used to fabricate hydrogels in various structures, and the structures of hydrogels have played diverse roles in bioengineering applications. We believe that the roles of hydrogel structures in bioengineering applications can be expanded through the development and diversification of SLA techniques.

On the other hand, there are limitations in the use of SLA processes to fabricate hydrogel structures. SLA processes generally illuminate hydrogel precursors to polymerize them. This can limit the types of hydrogel precursors that can be used for SLA processes to precursors that can be polymerized using light exposure [105,106,107]. Thus, natural polymers without inherent photo-crosslinking features need to have chemical modifications to be useful in SLA processes [87,108]. In addition, photo-initiators in the hydrogel precursors might cause cytotoxic effects during the polymerization of precursors [105,106,109]. The intensity, exposure time and wavelength of light and the type and concentration of photo-initiator can be considered to reduce the cytotoxic effects. Another challenge can be the balance between the shape fidelity and speed of the SLA processes [105,107,109]. In general SLA processes, small features close to the resolution of the system and with good shape fidelity can be obtained with a slow SLA printing speed, and increasing the speed might compromise shape fidelity. A relatively slow SLA printing speed is used for the fabrication of constructs with good printing fidelity or the large scale might cause dehydration of the fabricated constructs or a reduction of cell viability [106]. To overcome these challenges, strategies to improve the current techniques can be considered in future works. For instance, the types of hydrogel precursors can be expanded by developing novel photo-crosslinking polymers and hybrid materials exhibiting various functions [110]. Alternatively, SLA 3D printing systems can be improved to exert optimized control of light illumination and precursor flow for the rapid fabrication of hydrogel constructs.

## Figures and Tables

**Table 1:** Characteristics of SLA-, extrusion-, two-photon polymerization- and volumetric-type 3D printing systems.

Feature	SLA	Extrusion	Two-Photon Polymerization	Volumetric Printing
Resolution	High (10–100 µm)	Moderate (100–500 µm)	Very high (0.1–1 µm)	High (50–200 µm)
Printing speed	Moderate	Slow	Very slow	Fast
Precursor viscosity	Low	High	Low	Moderate
Cost efficiency	Moderate	Low	Very high	Moderate
Limitation in fabricable structure	Long overhanging structures can be limited	Support bath is required for overhanging structure printing	Thickness of constructs can be limited [37]	Structure stability can be limited
References	[35,40,41]	[33,34,42]	[36,37]	[43,44]

**Table 6:** Mechanical properties, degradation rates and cell viability of hydrogels used for nerve regeneration.

Hydrogel	Mechanical Properties	Degradation Rate	Cell Viability
(DBCO-GelMA [90]	Loss modulus: 19.98 Pa, tensile strength: 53.2 kPa	63.8 % mass loss in PBS at 37 °C over 21 days; 46.2% of the hydrogel remained after four weeks in a murine model	95.4% cell survival after 72 h
PGSm [58]	Young’s modulus: 3.2 MPa, elongation at break: 220 ± 14%	68.2% mass reduction in lipase-containting media after 72 h; 48.0% mass is left after 5 weeks of in vivo condition	94.8% cell survival after 72 h
GelMA/SF-MA [93]	Compressive strength: 16 N, tensile strength: 0.027–3.51 MPa depending on ratio	40.5% mass reduction after 14 days with Protease XIV; 49.3% of hydrogel remained after 4 weeks of in vivo condition	91.2% cell survival after 48 h

**Table 7:** Mechanical properties, degradation rates and cell viability of hydrogels used for bone regeneration.

Hydrogel	Mechanical Properties	Degradation Rate	Cell Viability
GelMA [60]	Young’s modulus: about 0.5 KPa	40% mass reduction over 14 days in collagenase	90.0% of cell survival after 72 h
GelMA/PEGDA/F-127 [65]	Compressive modulus: 92.34 ± 6.80 kPa, sweeling ratio 3.90 ± 0.62	57.81 ± 3.64% mass is left after 50 days	-
AAm/IBOA/WPUA [77]	Tensile strength: 9.9 MPa, elongation at break: 561–216%,	-	-

**Table 8:** Mechanical properties, degradation rates and cell viability of hydrogels used for skin and corneal regeneration.

Hydrogel	Mechanical Property	Degradation Rate	Cell Viability
GelMA/HA-NB [63]	Young’s modulus: 30.53 kPa	83% mass is retained after 4 h in PBS	95% of cell survival after 5 days
GelMA/Collagen [95]	Young’s modulus: 191 ± 35 kPa	-	96 ± 2% of cell survival ater 7 days
GelMA/CECM [69]	Storage modulus (G’): 4979.64 Pa, loss modulus (G’’): 132.66 Pa, Young’s modulus: 26.68 kPa: compressive strength of the hydrogel structure: 73.665 kPa	83% mass is retained after 4 h in PBS	94.84% of cell survival after 14 days
(NAGA)/GelMA [96]	Young’s modulus: 0.145 MPa, compressive strength: 6.9879 MPa	-	over 90% of cell survival after 7 days

**Table 9:** Mechanical properties, degradation rates and cell viability of hydrogels used for lab-on-a-chip devices.

Hydrogel	Mechanical Property	Degradation Rate	Cell Viability
GelMA/PEGDA [81]	Elastic modulus: 5.94 ± 0.19 kPa, storage modulus (G’): 2.07 ± 0.41 kPa	-	96 ± 2% of cell survival at 10 days
GelMA/PEGDA [80]	Fluid shear stress: 0.014–0.03 dyn/cm^2^	-	80 % on day 1, 70% on day 4
PEGDA/AAm [100]	Elastic modulus: from 45 kPa (soft matrix) to 210 kPa (stiff matrix)	No significant weight loss after 7 days	Over 90% viability at day 7.

**Table 10:** Mechanical properties, degradation rates and cell viability of hydrogels used for native tissue-like models.

Hydrogel	Mechanical Property	Degradation Rate	Cell Viability
GelMA/PEGDA [41]	Elastic modulus: below 8 kPa	28% mass reduction after 20 days	96 ± 2% of cell survival at 10 days
Sil-MA [104]	Compressive strength: 910 kPa, Compressive modulus: 125.8 kPa, Tensile strength: 75 kPa, Tensile modulus: 14.5 kPa	-	85–95% of cell survival

**Table 11:** Classification of hydrogel constructs used for bioengineering applications.

Application	Target Tissue	Structures	
Tissue regeneration	Sciatic Nerve [90,93]	Multi-layer, tube structures	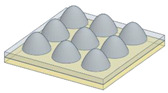 Multi-layer structure 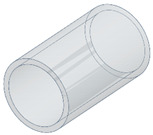 Tube structure 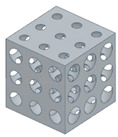 Lattice structure 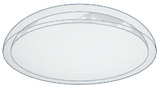 Dome structure
	Fibular nerve [58]	Multi-layer, tube structures	
	Parietal bone [60]	Multi-layer, lattice structures	
	Femoral bone [65]	Multi-layer, lattice structures	
	Cranial dorsal labrum and lateral meniscus [77]	Multi-layer structure	
	Skin defects [63,95]	Multi-layer, lattice structures	
	Corneal stroma [69,96]	Multi-layer, dome structures	
Lab-on-a-chip devices	Intestinal tissue model [80,81]	Multi-layer, lattice structures	
	Hepatic lobule [100]	Multi-layer, channel	

## Data Availability

No new data were created or analyzed in this study. Data sharing is not applicable to this article.
